# The palatine tonsil bacteriome, but not the mycobiome, is altered in HIV infection

**DOI:** 10.1186/s12866-018-1274-9

**Published:** 2018-10-05

**Authors:** Yuto Fukui, Kotaro Aoki, Yoshikazu Ishii, Kazuhiro Tateda

**Affiliations:** 1 Department of Microbiology and Infectious Diseases, Toho University Graduate School of Medicine, 5-21-16 Omorinishi, Ota-ku, Tokyo, 143-8540 Japan; 2 Department of Infectious Diseases, Toho University Omori Medical Center, 6-11-1 Omorinishi, Ota-ku, Tokyo, 143-8541 Japan

**Keywords:** Human immunodeficiency virus, Palatine tonsil microbiome, Bacteriome, Mycobiome

## Abstract

**Background:**

Microbial flora in several organs of HIV-infected individuals have been characterized; however, the palatine tonsil bacteriome and mycobiome and their relationship with each other remain unclear. Determining the palatine tonsil microbiome may provide a better understanding of the pathogenesis of oral and systemic complications in HIV-infected individuals. We conducted a cross-sectional study to characterize the palatine tonsil microbiome in HIV-infected individuals.

**Results:**

Palatine tonsillar swabs were collected from 46 HIV-infected and 20 HIV-uninfected individuals. The bacteriome and mycobiome were analyzed by amplicon sequencing using Illumina MiSeq. The palatine tonsil bacteriome of the HIV-infected individuals differed from that of HIV-uninfected individuals in terms of the decreased relative abundances of the commensal genera *Neisseria* and *Haemophilus*. At the species level, the relative abundances and presence of *Capnocytophaga ochracea*, *Neisseria cinerea*, and *Selenomonas noxia* were higher in the HIV-infected group than those in the HIV-uninfected group. In contrast, fungal diversity and composition did not differ significantly between the two groups. Microbial intercorrelation analysis revealed that *Candida* and *Neisseria* were negatively correlated with each other in the HIV-infected group. HIV immune status did not influence the palatine tonsil microbiome in the HIV-infected individuals.

**Conclusions:**

HIV-infected individuals exhibit dysbiotic changes in their palatine tonsil bacteriome, independent of immunological status.

**Electronic supplementary material:**

The online version of this article (10.1186/s12866-018-1274-9) contains supplementary material, which is available to authorized users.

## Background

Human immunodeficiency virus (HIV) infection is characterized by progressively reduced cell-mediated immunity, as reflected by the CD4^+^ T-cell count, resulting in increased opportunistic infections [1]. Antiretroviral therapy (ART) has increased the life expectancy of HIV-infected individuals, accompanied by improved cellular immunity and reduced frequency of diseases associated with acquired immunodeficiency syndrome (AIDS) [2, 3]. Despite the successful use of ART, HIV-infected individuals continue to experience excessive morbidity and mortality from non-AIDS defining infectious diseases and noninfectious chronic comorbidities [4, 5].

The microbiome (the collective genomes of microbial flora) of HIV-infected individuals is gradually being elucidated [6, 7], as human microbiome research progresses [8]. The role of microbial flora in pathogenesis of HIV infection has been the subject of much research in recent years [7, 9–13]. Dysbiosis during HIV infection such as increased abundance of pathogenic *Prevotella* in the oral cavity and decreased abundance of *Lactobacillus* in the gut has been reported, and the loss of a normal microbial flora may influence HIV transmission, prevention, progression and prognosis [12, 14–20]. However, most studies that have characterized the human microbiome in health and disease, including in HIV infection, have focused only on the bacteriome [21]. Fungi are also important in the human microbiome [22]; thus, more research is needed on the mycobiome and its relationships with the bacteriome.

The human palatine tonsils are mucosa-associated lymphoid tissues (MALT) located in the oropharynx and provide mucosal protection [23]. Based on their location, the palatine tonsils are among the early sites that encounter microbial and environmental antigens in the human body [23, 24]. Therefore, the palatine tonsil microbiome of HIV-infected individuals may play a key role in opportunistic infections; however, this remains to be investigated.

We conducted a cross-sectional study to characterize the palatine tonsil bacteriome, mycobiome and their intercorrelations with each other among HIV-infected individuals.

## Results

### Participants characteristics

Forty-six HIV-infected and 20 HIV-uninfected participants were enrolled. No differences in age, sex, smoking status, or white blood cell (WBC) count were noted between the groups (Table 1). No individuals had severe periodontal diseases that required treatment. The median and nadir CD4^+^ T-cell count of the HIV-infected group was 438 cells/μL (interquartile range [IQR] 274–618) and 164 cells/μL (IQR 94–306), including 6 severely immunocompromised individuals (13%) with CD4^+^ T-cell counts below 200 cells/μL. The median plasma viral load of the HIV-infected group was 0 copy/mL (IQR 0–4505), with 13 individuals having detectable viremia (viral load > 200 copies/mL). Thirty-four of the 46 HIV-infected individuals (74%) received ART.

**Table 1:** Study participant characteristics

	HIV-infected *N* = 46	HIV-uninfected *N* = 20	*p* value
Age, mean (SD), years	41 (13)	40 (10)	0.57
Gender (male), *n* (%)	43 (94)	18 (90)	0.64
Smoker, *n* (%)	16 (35)	9 (45)	0.26
Risk behaviors for HIV infection			
MSM, *n* (%)	38 (83)	NA	ND
IDU, *n* (%)	5 (11)	NA	ND
WBC, mean (SD), count/dL	5424 (1623)	5995 (1682)	0.20
CD4^+^ T-cell count, median cells/μL (IQR)	438 (274–618)	ND	ND
Nadir CD4^+^ T-cell count, median cells/μL (IQR)	164 (94–306)	ND	ND
CD4^+^ T-cell count ≦ 200 cells/μL, *n* (%)	6 (13)	ND	ND
Plasma viral load, median copies/mL (IQR)	0 (0–4505)	ND	ND
Plasma viral load > 200 copies/mL, *n* (%)	13 (28)	ND	ND
Treatment with ART, *n* (%)	34 (74)	NA	ND

*SD* standard deviations, *MSM* men who have sex with men, *IDU* intravenous drug users, *NA* not applicable, *WBC* white blood cell, *IQR* interquartile range, *ND* not done, *ART* antiretroviral therapy

Age and WBC counts were summarized by means and compared using *t*-tests. Gender and smoking status were compared using Fisher’s exact tests

### HIV-infected individuals had an altered palatine tonsil bacteriome

One HIV-infected sample did not contain sufficient reads for analysis; therefore, 45 HIV-infected and 20 HIV-uninfected participants were included in the bacterial analysis. We first assessed the alpha diversity (the number of observed operational taxonomy units [OTUs], Shannon entropy and phylogenetic diversity) of the palatine tonsil bacteriome from 14,737 reads. The HIV-infected individuals had a significantly increased number of observed OTUs (Additional file 1: Table S1). Beta diversity, determined by principal coordinates analysis using weighted UniFrac distance metrics, showed that the HIV-infected palatine tonsil bacteriome was distinct from that of the HIV-uninfected individuals (*p* = 0.02, permutational multivariate analysis of variance [PERMANOVA]) (Additional file 2: Figure S1).

To determine which taxa differed between the HIV-infected and HIV-uninfected individuals, we compared the relative abundances between them at different taxonomic levels.

Phylum-level analysis revealed that five phyla (*Firmicutes*, *Bacteroidetes*, *Proteobacteria*, *Fusobacteria*, *Actinobacteria*) were abundant, and *Firmicutes* was the most abundant in both groups (Additional file 1: Table S2). Compared to the HIV-uninfected individuals, the HIV-infected individuals had a significantly increased relative abundance of *Firmicutes* (*p* = 0.009) and significantly decreased relative abundances of *Proteobacteria* (*p* = 0.04) and *Fusobacteria* (*p* = 0.03).

**Fig. 1:**
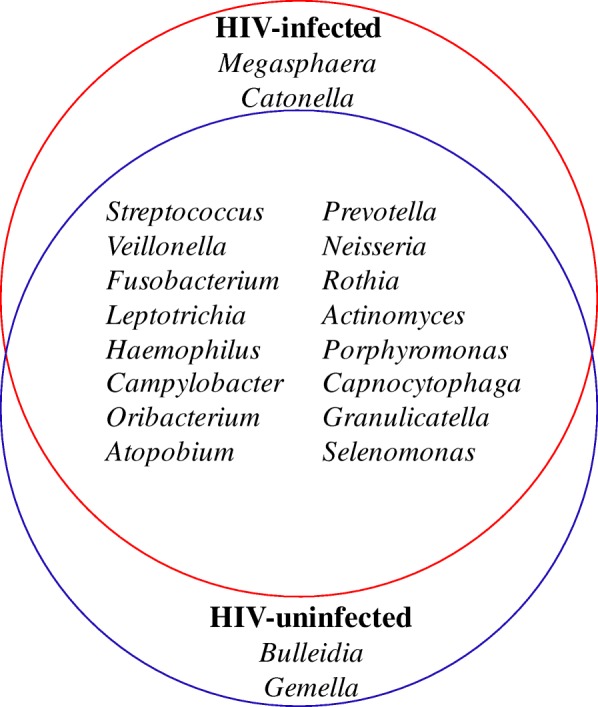
Palatine tonsil core bacteriome in the HIV-infected and HIV-uninfected individuals

**Fig. 2:**
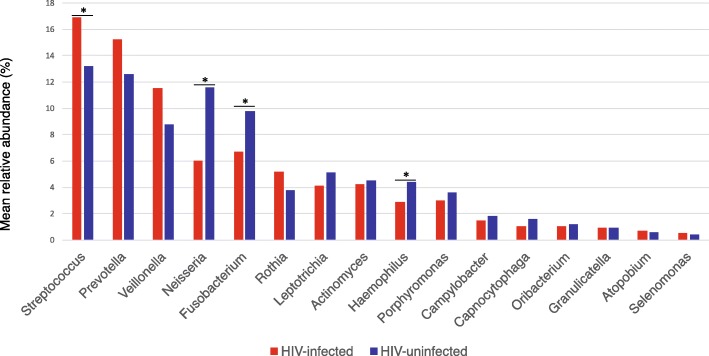
Relative abundances of the common core bacterial genera in the HIV-infected and HIV-uninfected individuals; *p <  0.05, Wilcoxon rank-sum test

A total of 82 genera were detected in both groups. At the genus level, we identified the core bacteriome of each group, which was defined as the genera present in 100% of samples [25]. The core palatine tonsil bacteriome consisted of 18 genera in both groups, of which 16 genera (*Streptococcus*, *Prevotella*, *Veillonella, Neisseria, Fusobacterium, Rothia, Leptotrichia, Actinomyces, Haemophilus, Porphyromonas, Campylobacter, Capnocytophaga, Oribacterium, Granulicatella, Atopobium, Selenomonas*) were common to both groups (Fig. 1). *Megasphaera* and *Catonella* belonged to the core bacteriome of the HIV-infected group, and *Bulleidia* and *Gemella* belonged to that of the HIV-uninfected group. Comparing the relative abundance of the common core bacteriome genera, we found that *Neisseria* (6% mean relative abundance for the HIV-infected individuals and 11.2% for the HIV-uninfected individuals, *p* = 0.03), *Fusobacterium* (6.7% and 9.8%, respectively, *p* = 0.02) and *Haemophilus* (2.9% and 4.4%, respectively, *p* = 0.01) were significantly decreased, and *Streptococcus* (16.9% and 13.2%, respectively, *p* = 0.04) was significantly increased in the HIV-infected group (Fig. 2).

To identify major species-level differences, we searched the species whose core bacteriome colonization rate or relative abundance differed significantly between the groups. *Capnocytophaga ochracea*, *Neisseria cinerea*, and *Selenomonas noxia* colonized the HIV-infected group more frequently, with a higher relative abundance than that in the HIV-uninfected group (Table 2). *Veillonella parvula* had a similar colonization rate, but its relative abundance was significantly higher in the HIV-uninfected group than that in the HIV-infected group (Table 2).

**Table 2:** Bacterial species with different presence and relative abundances in the HIV-infected and HIV-uninfected individuals

	Presence (%)			Median relative abundance, %		
	HIV-infected *N* = 45	HIV-uninfected *N* = 20	*p* value	HIV-infected *N* = 45	HIV-uninfected *N* = 20	*p* value
*Capnocytophaga ochracea*	26 (58)	1 (5)	< 0.001	0.01	0	< 0.001
*Neisseria cinerea*	24 (53)	4 (20)	0.02	0.07	0	0.005
*Selenomonas noxia*	28 (62)	5 (25)	0.007	0.01	0.0001	0.02
*Veillonella parvula*	44 (98)	19 (95)	0.52	0.3	2.0	0.01

Presence was compared using Fisher’s exact tests. Relative abundance was summarized by medians and compared using Wilcoxon rank-sum tests

### The palatine tonsil mycobiome did not differ between HIV-infected and HIV-uninfected individuals

One HIV-infected sample and three HIV-uninfected samples did not yield sufficient reads for analysis; therefore, 45 HIV-infected and 17 HIV-uninfected participants were included in the fungal analysis. We first assessed the alpha diversity of the palatine tonsil mycobiome in 52 reads. Unlike the bacteriome, the HIV-infected and HIV-uninfected samples did not significantly differ in terms of alpha diversity (Additional file 1: Table S3). Beta diversity, determined by principal coordinates analysis using weighted UniFrac distance metrics, also did not differ between the two groups (*p* = 0.47, PERMANOVA) (Additional file 3: Figure S2).

We compared the relative abundances between the two groups at different taxonomic levels to determine the impact of HIV infection on the palatine tonsil mycobiome.

Phylum-level analysis revealed that two phyla, *Ascomycota* and *Basidiomycota*, were abundant, with *Ascomycota* being the most abundant in both groups (Additional file 1: Table S4). The relative abundances of *Ascomycota* and *Basidiomycota* did not significantly differ between the groups. Of the total reads, 1.5% were classified as unidentified phyla or not classified.

**Fig. 3:**
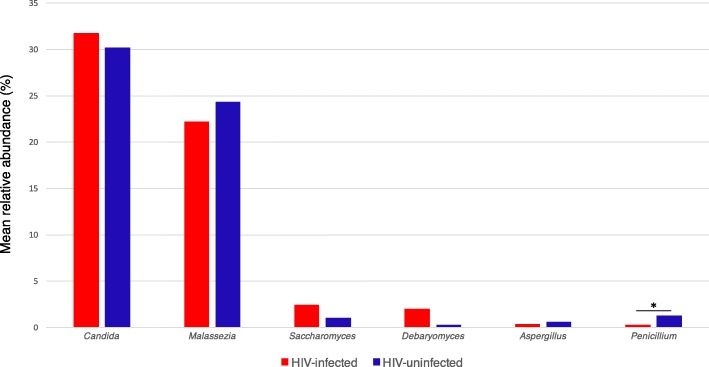
Relative abundances of the six most abundant fungal genera in the HIV-infected and HIV-uninfected individuals; *p <  0.05, Wilcoxon rank-sum test

Genus-level analysis identified a total of 43 genera in both groups. The median number of detected genera did not significantly differ between the HIV-infected (4, IQR 4–6) and HIV-uninfected individuals (5, IQR 4–6) (*p* = 0.6). The six most abundant fungal genera were *Candida*, *Malassezia*, *Saccharomyces*, *Debaryomyces*, *Aspergillus* and *Penicillium* (Fig. 3). *Candida* and *Malassezia* colonized the majority of both groups, but no genera colonized 100% of each group. Only the relative abundance of *Penicillium* was significantly higher in the HIV-uninfected individuals than that in the HIV-infected individuals among the six most abundant genera.

We used linear discriminant effect size analysis (LEfSe) with default parameters to further identify fungal taxa that were differentially represented between the HIV-infected and HIV-uninfected individuals [26]. LEfSe analysis revealed that the HIV-uninfected group had a significant increase in the order *Eurotiales* and its family *Trichocomaceae*, which contains the genera *Aspergillus* and *Penicillium* (Additional file 4: Figure S3). No fungal OTUs differed significantly between the groups by LEfSe.

### Microbial intercorrelations differed in the HIV-infected individuals

We determined the correlations between individual genera of the palatine tonsil bacteriome and mycobiome within their respective communities and across the two communities. In the core bacteriome, the correlation pattern differed between the groups. The HIV-infected group had 13 positive bacterial relationships and 9 negative relationships (Additional file 1: Table S5), while the HIV-uninfected group had 7 positive bacterial relationships and 5 negative relationships (Additional file 1: Table S6). In the mycobiome, only HIV-infected group had 1 negative relationship (*Candida-Malassezia*, rho = − 0.56, *p* <  0.001) among the six most abundant genera. Next, we assessed the correlation between the bacteriome and the mycobiome. The numbers of observed bacterial and fungal OTUs did not correlate with each other in either the HIV-infected (rho = − 0.01, *p* = 0.95) or HIV-uninfected groups (rho = − 0.15, *p* = 0.55) (Additional file 5: Figure S4). The HIV-infected group had no strong correlations (rho > 0.5 or rho < − 0.5), and 3 weak correlations (rho > 0.3 or rho < − 0.3) between the bacteriome and mycobiome (Additional file 1: Table S7). In comparison, the HIV-uninfected group had 2 strong correlations (Additional file 1: Table S8). Both groups demonstrated negative relationships between *Candida* and *Neisseria* and *Candida* and *Capnocytophaga.*

### HIV immune status did not impact the palatine tonsil microbiome in HIV-infected individuals

We studied the relationship between CD4^+^ T-cell counts and the microbiome in the HIV-infected individuals. CD4^+^ T-cell counts were not correlated with the number of observed bacterial (rho = 0.06, *p* = 0.69) or fungal (rho = 0.21, *p* = 0.17) OTUs (Additional file 6: Figure S5). When the HIV-infected individuals were divided into a high CD4 (CD4^+^ T-cell count ≥350 cells/μ) or low CD4 group (CD4^+^ T-cell count < 350 cells/μ), the two groups did not significantly differ in terms of their alpha diversity of bacteriome (Additional file 1: Table S9) and mycobiome (Additional file 1: Table S10). Beta diversity by principal coordinates analysis using weighted UniFrac distance metrics also did not differ between the two groups (bacteriome, *p* = 0.76; mycobiome, *p* = 0.92, PERMANOVA). We did not observe any influence of different HIV immune status (ART-treated vs ART-untreated and detectable viremia vs no viremia) on bacteriome and mycobiome in terms of alpha and beta diversity (data not shown). We also compared the microbiome between Men who have sex with men (MSM) and non-MSM and did not observe the difference between them (data not shown).

## Discussion

Our study identified the palatine tonsil core microbiome and showed that the HIV-infected individuals had a different palatine tonsil bacteriome and bacterial intercorrelations than the HIV-uninfected individuals. In contrast, the palatine tonsil mycobiome did not differ significantly between the two groups. The microbial intercorrelations between the bacteriome and mycobiome were relatively similar between the two groups. In the HIV-infected individuals, HIV immune status did not impact the palatine tonsil microbiome, suggesting that HIV infection itself played an important role in palatine tonsil microbial composition.

As with previous studies that analyzed the bacteriome of other specimens from HIV-infected individuals, such as saliva, bronchoalveolar lavage (BAL) and stool [6, 7, 13, 27], the HIV-infected individuals had altered palatine tonsil bacteriome. Changes of alpha diversity in HIV infection are variable, increased in some studies, decreased or not changed in others [20, 27–31]. However, most previous cross-sectional studies detected dysbiotic changes, such as the emergence and increase in pathogenic bacteria and decrease in beneficial bacteria, in HIV-infected individuals [7]. Alterations of bacterial composition in HIV infection differ among organs. We observed increase of *Firmicutes* and decrease of *Proteobacteria* in the palatine tonsil. However, previous studies reported inverse results in the gut and the mouth, and others did not note a difference among these phyla in the rectum [11, 15, 32]. This may be because each organ has a specific microbiome and is influenced differently by HIV infection. In the oral microbiome of HIV-infected individuals, the relative abundances of *Prevotella*, *Megasphaera* and *Campylobacter* increased, while that of the normal oral flora *Streptococcus* decreased [14, 33]. Another study reported that the genus *Capnocytophaga* is a member of the core oral microbiome in HIV-infected individuals [21] and is associated with AIDS patients with periodontitis [34]. In our study, we observed the genus *Capnocytophaga* to be the common core microbiome in both the HIV-infected and HIV-uninfected individuals. However, the relative abundance and presence of *Capnocytophaga ochracea* increased significantly in the palatine tonsil microbiome of the HIV-infected individuals, regardless of immune status. *C. ochracea* is reported to produce an immunosuppressive factor [35] and to degrade immunoglobulin [36]. These abilities may enhance colonization of this or other opportunistic pathogens and may be involved in the development of opportunistic respiratory infections by modulating the immune system and inducing dysbiosis in HIV-infected individuals. Further research is needed to determine the effect of *C. ochracea* on HIV-infected individuals.

In contrast to the bacteriome, the palatine tonsil mycobiome demonstrated only small compositional differences and did not differ in alpha or beta diversity between the two groups. Few studies have been published on the oral and respiratory mycobiome of HIV-infected individuals [21, 37]. A previous mycobiome research on oral wash samples found that the core mycobiome of HIV-infected individuals was different from that of controls [21]. However, this study compared only some fungal composition and did not compare alpha or beta diversity; therefore, overall mycobiome differences between HIV-infected and HIV-uninfected individuals were unclear. *Cryptococcus* species and *P*. *jirovecii*, which are frequent opportunistic pathogens [38–40] and are identified as part of the lung mycobiome in HIV-infected individuals [37], were not detected in the palatine tonsil mycobiome. This result indicated that each organ has a distinct community structure and the palatine tonsil may not be an important organ for opportunistic fungal colonization.

The palatine tonsillar bacterial community in HIV-infected individuals differed in its taxonomic composition as well as its bacterial genera correlation patterns. For example, the negative relationship between the normal commensal flora *Neisseria* [41] and the pathogenic genus *Prevotella* [42] was lost in HIV-infected individuals; however, *Neisseria*-*Veillonella* were negatively correlated and *Prevotella*-*Veillonella* were positively correlated in HIV-infected groups. Decreased *Neisseria* resulted in increased *Prevotella* and *Veillonella* in HIV-infected individuals. Increased numbers of *Prevotella* and *Veillonella* have been shown to be associated with respiratory inflammation [42]. Another study indicated that increased *Prevotella* abundance in HIV-infected individuals was related to mucosal and systemic immune activation [32]. Chronic inflammation induced by an altered bacteriome may lead to non-AIDS-defining oral diseases, such as oral squamous cell carcinoma, recurrent aphthous ulcers and necrotizing ulcerative periodontitis, in HIV-infected individuals.

Although there were slight differences, the microbial intercorrelations between the bacteriome and mycobiome were relatively similar between both groups. Both demonstrated negative correlations between *Neisseria* and *Candida* and *Capnocytophaga* and *Candida*. These results suggest that universal relationships between *Candida* and symbiotic bacteria may inhibit or support each other within the pharyngeal microbiome. Previous research showed that commensal bacterial species inhibit *Candida* virulence and growth by preventing biofilm formation and hyphenation [43–46]. In addition, several commensal bacteria have been described to modulate the murine immune system via Th17 cells and Treg cells; these cells are essential for the immune response against *Candida* [47–49]. In practice, specific medical conditions, such as antibiotic use and intensive care unit stays, may cause human microbiome dysbiosis [50, 51] and increase *Candida* infections [52]. We speculate that dysbiotic microbial flora and increased candidiasis in HIV-infected individuals may be correlated.

The influence of immune status on the microbiome remains unclear in HIV-infected individuals. A recent study reported that CD4^+^ T-cell counts do not affect oral or BAL microbiome in HIV-infected individuals [33]. Another study showed that introduction of ART has no influence on the oral microbiome [13]. Despite immune status recovery due to ART, microbiome and metagenomic functions in HIV-infected individuals have been found to differ from those of healthy individuals, suggesting HIV infection itself is important to the microbiome [28, 29, 53]. HIV-infected individuals are more likely to develop cardiovascular disease, lung disease and malignancy, even in the ART era [54–56]. A dysbiotic microbiome is considered to play a prominent role in increased comorbidities in HIV-infected individuals [57].

This study had several limitations. First, it focused on bacterial and fungal communities, but other organisms, such as viruses, archaea and protozoa, may also be factors [58]. Another limitation is that some factors such as diet, risk behaviors and antibiotics use in past months were not controlled in our analysis. Finally, we only included a few HIV-infected individuals with severe immunodeficiency (CD4^+^ T-cell count < 200 cells/μL or ART-untreated), due to the exclusion criterion of antibiotic use. Therefore, more HIV-infected individuals with severe immunodeficiency are needed to elucidate the true impact of HIV immune status on the microbiome.

## Conclusion

We conducted a cross-sectional study to describe the palatine tonsil microbiome in HIV-infected individuals and found that the bacteriome and bacterial intercorrelations differed significantly between the HIV-infected and HIV-uninfected individuals. In contrast to the bacteriome, the palatine tonsil mycobiome did not differ significantly between the two groups. The microbial intercorrelations between the bacteriome and mycobiome were relatively similar between both groups. In the HIV-infected individuals, HIV immune status did not impact the palatine tonsil microbiome, suggesting that HIV infection itself played a vital role in the palatine tonsil microbial composition.

## Methods

### Participants and sample collection

During the study period between October 2016 and June 2017, 72 HIV-infected individuals from Toho University Omori Medical Center were initially evaluated. Exclusion criteria were: (a) having acute respiratory symptoms; (b) taking more than five medications besides ART; (c) antibiotics use within the last 4 weeks; (d) history of palatine tonsillectomy; (e) unable to be sampled correctly for anatomical reasons; (f) disagreement to participate. We excluded total 26 HIV-infected individuals (14 with [b], 6 with [f], 5 with [c] and 1 with [e]). Forty-six participants were included after meeting the criteria. Additionally, 20 sex- and age-matched healthy HIV-negative individuals were included from the same medical center. We obtained clinical and demographic data by performing standardized subject interviews and medical record reviews. This cross-sectional study protocol was approved by the Institution Ethics Committee of Toho University School of Medicine (Number: A16072_A17082). All participants provided written informed consent in accordance with the Declaration of Helsinki. Palatine tonsil microbiome samples were collected with Catch-All Sample Collection Swabs (Epicentre, Madison, WI, USA) following Human Microbiome Project procedures (http://hmpdacc.org/doc/HMP_MOP_Version12_0_072910.pdf). Before sampling, mouthwashes were not used. Immediately after swabbing, each swab was swirled in 750 μL of MoBio buffer in a MoBio tube (MO BIO Laboratories, Inc., Carlsbad, CA, USA) on ice, and the tubes were stored at − 80 °C until DNA extraction was performed. All specimens were taken by a single doctor to avoid sampling biases.

### DNA extraction and sequencing

All samples, including negative control swab samples, were collected, and genomic DNA (gDNA) was extracted using a PowerSoil DNA Isolation Kit (MO BIO Laboratories, Inc., Carlsbad, CA, USA), with minor modifications [59]. Briefly, samples were heat-lysed at 70 °C for 10 min after adding C1 lysis buffer. The mechanical bead beating step was performed using FastPrep FP120 (Thermo Savant, Carlsbad, CA, USA) for 90 s at 5.5 speed to enhance chemical, heat, and mechanical lysis. A blank swab was included as a control in the gDNA extraction step to assess possible contamination.

Sequencing libraries were prepared for Illumina MiSeq (Illumina, San Diego, CA, USA). For bacterial analysis, the primer set consisting of 341F (5′-CCTACGGGNGGCWGCAG-3′) and 806R (5′-GACTACHVGGGTATCTAATCC-3′) was used to target the 16S rRNA V3 and V4 regions [60]. A 16S rRNA library for the Illumina MiSeq platform was prepared per the manufacturer’s protocol (http://jp.support.illumina.com/content/dam/illumina-support/documents/documentation/chemistry_documentation/16s/16s-metagenomic-library-prep-guide-15044223-b.pdf). In the PCR clean-up step, a Wizard SV Gel and PCR Clean-Up System (Promega, St. Louis, MO, USA) was used to purify PCR amplicons [61]. For fungal analysis, we amplified the internal transcribed spacer (ITS) 1 rDNA region with a primer set consisting of ITS1F (5′-CTTGGTCATTTAGAGGAAGTAA-3′) and ITS2 (5′- GCTGCGTTCTTCATCGATGC-3′) [21]. DNA was amplified with an initial denaturation step at 95 °C for 3 min, followed by 40 cycles of denaturation at 95 °C for 30 s, annealing at 55 °C for 30 s, and elongation at 72 °C for 30 s, followed by a final elongation step at 72 °C for 5 min. Duplicate amplifications were performed for each reaction and mixed before the PCR clean-up step. The subsequent procedure was the same as that used for the bacterial analysis. Each library was sequenced with 2 × 300-bp paired-end reads on a MiSeq system using MiSeq v3 reagent kits (Illumina, San Diego, CA, USA).

### Sequence analysis and statistical analysis

MiSeq sequencing resulted in a total of 4,169,518 reads for the 16S rRNA with a mean of 63,175 ± 18,146 sequences per sample and 4,145,030 reads for the ITS1 region with a mean of 62,803 ± 11,681 sequences per sample. Sequence data were submitted to DDBJ under the accession number DRA006313.

The sequencing data were processed using CLC Genomic Workbench 10.0.1 and CLC Microbial Genomics Module 2.5 (Qiagen, Redwood City, CA, USA) [62]. The overlapping paired-end reads were merged and trimmed, and chimeric reads were filtered using default parameters. The remaining reads were clustered into OTUs with 97% identity using the Greengenes database (version 13_5) as the reference for the 16S rRNA data [63] and the UNITE database (version 7.1) as the reference for the ITS data [64]. OTUs with less than 42 reads were removed from bacterial analysis, leaving a total of 511 bacterial OTUs. OTUs annotated to kingdom Plantae and sequences detected in the control were removed from fungal analysis, leaving a total of 238 fungal OTUs.

Alpha diversity was calculated as the number of observed OTUs, Shannon entropy [65] and phylogenetic diversity [66] by CLC Genomic Workbench. Beta diversity was measured as a weighted UniFrac distance based on the OTU table [67] by CLC Genomic Workbench. Relative abundance of the taxa was calculated from an unrarefied OTU table. We used *t*-tests to compare continuous variable means, Fisher’s exact tests to compare categorical variable proportions and the Wilcoxon rank-sum test to compare alpha diversity and relative abundance values between the groups using R [68]. PERMANOVA was performed to compare beta diversity using CLC Genomic Workbench. Spearman’s correlation tests were computed using R and illustrated with the *qgraph* R package [69]. LEfSe, which is an algorithm for identifying genomic taxa whose relative abundances differ significantly between groups [26], was used in the mycobiome analysis with default parameters. *p* values < 0.05 were considered to indicate statistical significance. *p* values were corrected for multiple testing controlling the false discovery rate at analyzing the intercorrelations [70].

## Additional files

Additional file 1: **Table S1.** Bacterial alpha diversity of the palatine tonsil. **Table S2.** Bacterial phyla and their differences in relative abundance (mean, %) between HIV-infected and uninfected individuals in the palatine tonsil. **Table S3.** Fungal alpha diversity of the palatine tonsil. **Table S4.** The relative abandance (mean, %) of fungal phyla in the palatine tonsil. **Table S5.** Correlations among the common core bacterial genera in HIV-infected individuals. **Table S6.** Correlations among the common core bacterial genera in HIV-uninfected individuals. **Table S7.** Correlation between bacteriome and mycobiome in HIV-infected individuals. **Table S8.** Correlation between bacteriome and mycobiome in HIV-uninfected individuals. **Table S9.** Bacterial alpha diversity of palatine tonsil in HIV-infected individuals. **Table S10.** Fungal alpha diversity of palatine tonsil in HIV-infected individuals. (XLSX 21 kb)

Additional file 2: **Figure S1.** Principal coordinates analysis plots of bacterial beta diversity using weighted UniFrac distance. The HIV-infected and HIV-uninfected individuals are colored red and blue, respectively. (PDF 50 kb)

Additional file 3: **Figure S2.** Principal coordinates analysis plots of fungal beta diversity using weighted UniFrac distance. The HIV-infected and HIV-uninfected individuals are colored red and blue, respectively. (PDF 47 kb)

Additional file 4: **Figure S3.** LEfSe shown as a cladogram (a) and LDA score (b). The HIV-uninfected individuals are displayed in red. (PDF 224 kb)

Additional file 5: **Figure S4.** Scatter plot showing the relationship between the number of observed bacterial and fungal OTUs in the HIV-infected (a) and HIV-uninfected individuals (b). (PDF 67 kb)

Additional file 6: **Figure S5.** Scatter plot showing the relationship between CD4+ T-cell counts and the number of observed bacterial (a) and fungal OTUs (b) in the HIV-infected individuals. (PDF 57 kb)

## Abbreviations

AIDS: Acquired immunodeficiency syndrome
ART: Antiretroviral therapy
BAL: bronchoalveolar lavage
gDNA: Genomic DNA
HIV: Human immunodeficiency virus
IQR: Interquartile range
ITS: Internal transcribed spacer
LEfSe: Linear discriminant effect size analysis
MALT: Mucosa-associated lymphoid tissue
MSM: Men who have sex with men
NA: Not applicable
ND: Not done
OTU: Operational taxonomy unit
PERMANOVA: Permutational multivariate analysis of variance
SD: Standard deviations
WBC: White blood cell
