# Supplementary figures and images for: The palatine tonsil bacteriome, but not the mycobiome, is altered in HIV infection
Source: BMC Microbiol. 2018 Oct 5;18:127. doi: 10.1186/s12866-018-1274-9 (PMC6173881; doi:10.1186/s12866-018-1274-9)

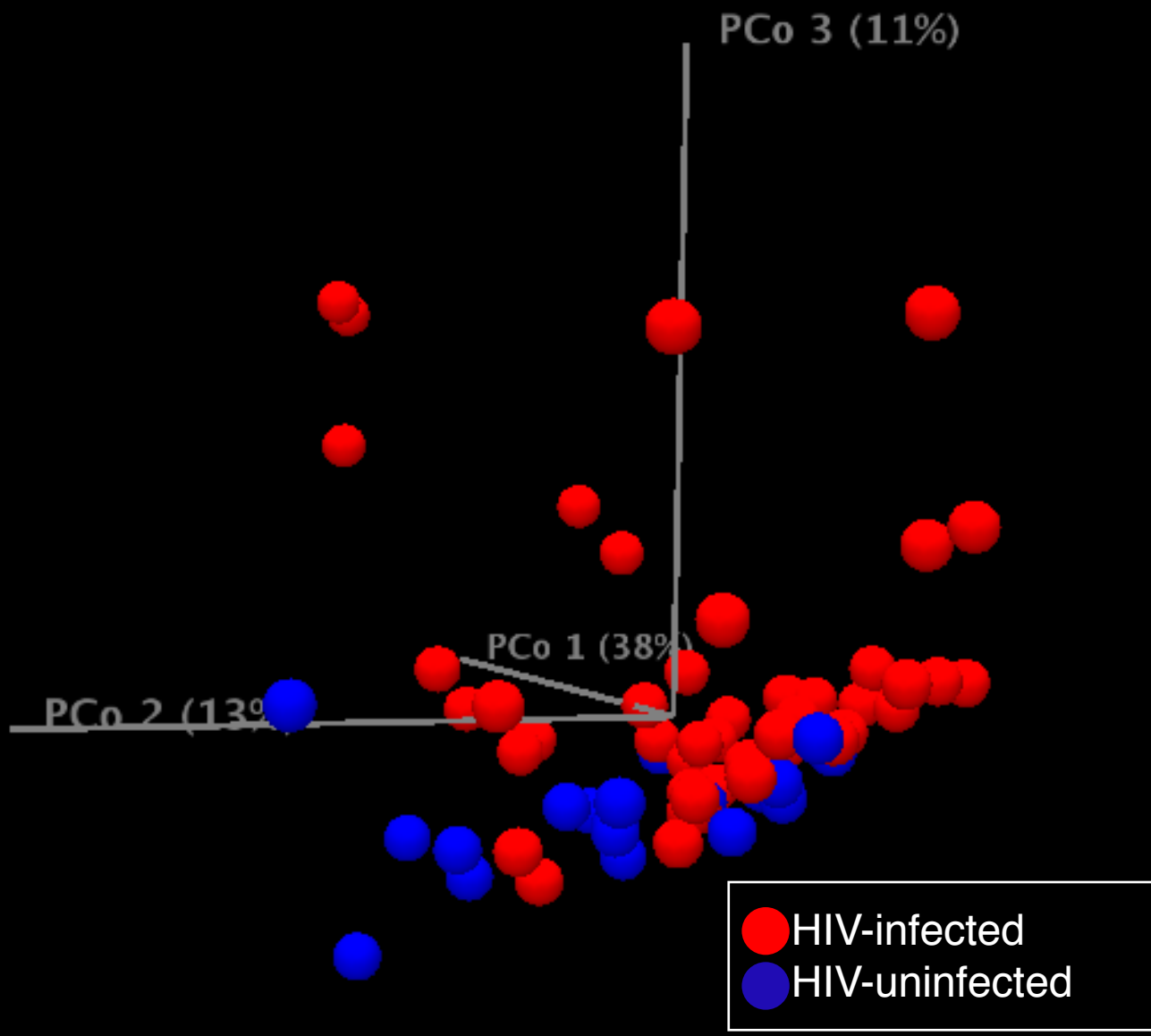

Supplement: Supplementary file 2 — Figure S1. Principal coordinates analysis plots of bacterial beta diversity using weighted UniFrac distance. The HIV-infected and HIV-uninfected individuals are colored red and blue, respectively. (PDF 50 kb) [file 12866_2018_1274_MOESM2_ESM.pdf]

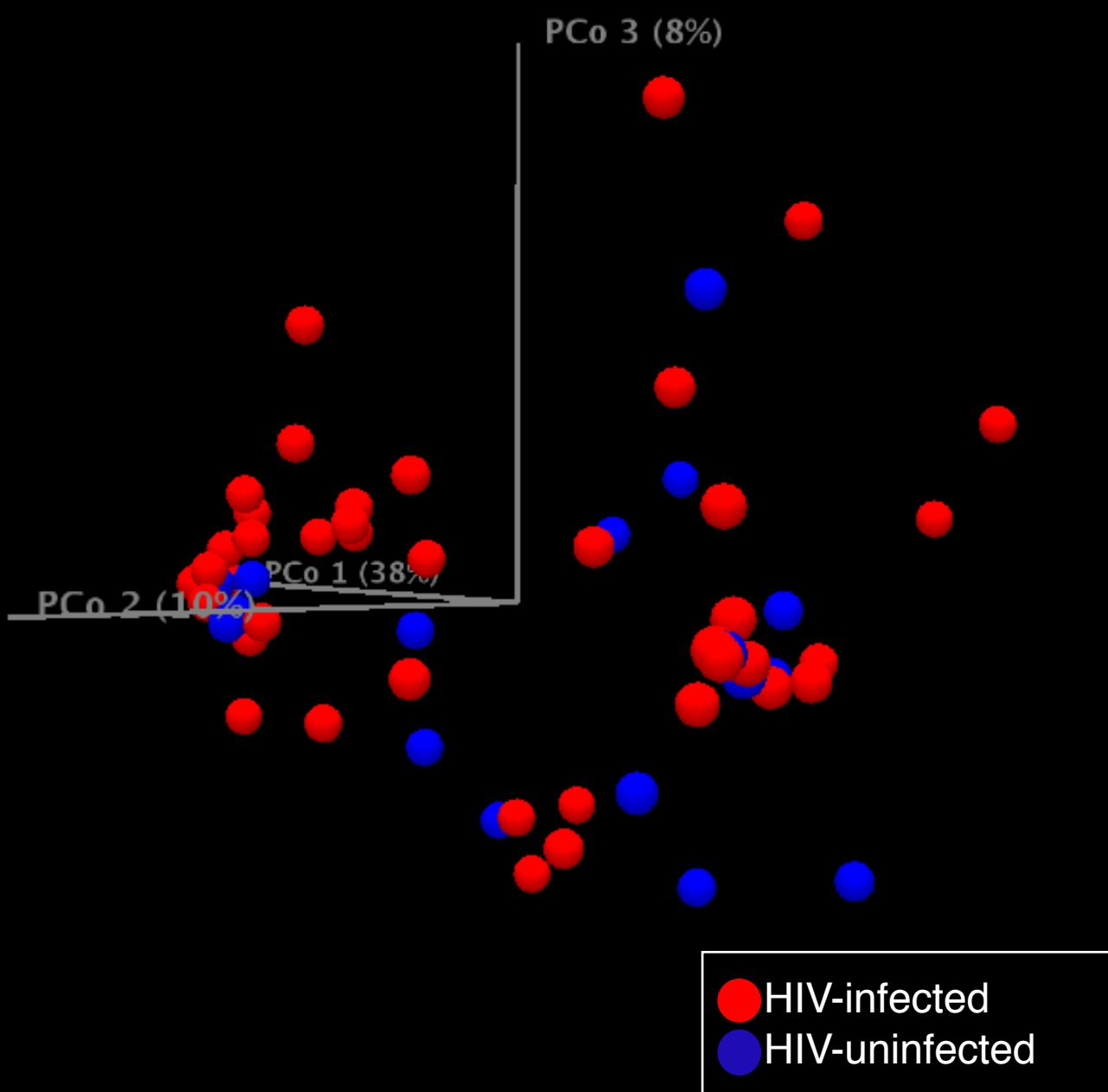

Supplement: Supplementary file 3 — Figure S2. Principal coordinates analysis plots of fungal beta diversity using weighted UniFrac distance. The HIV-infected and HIV-uninfected individuals are colored red and blue, respectively. (PDF 47 kb) [file 12866_2018_1274_MOESM3_ESM.pdf]

**a**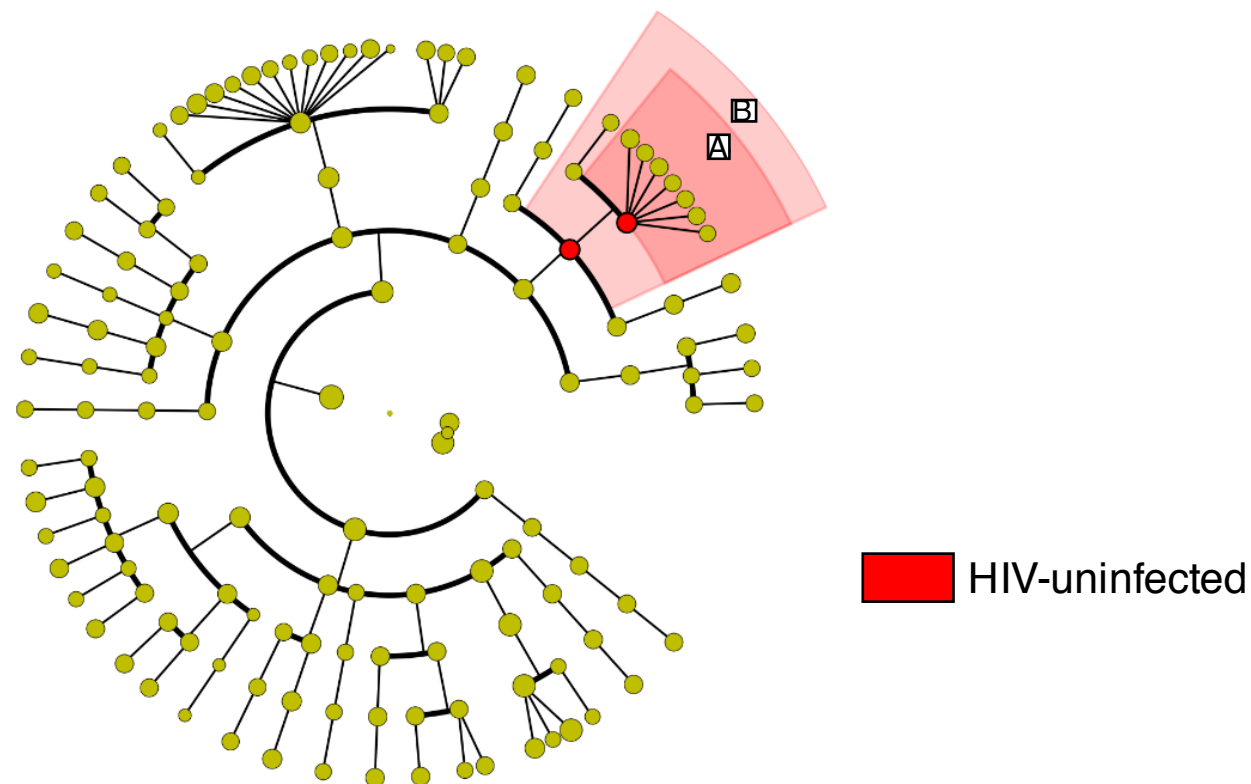**b**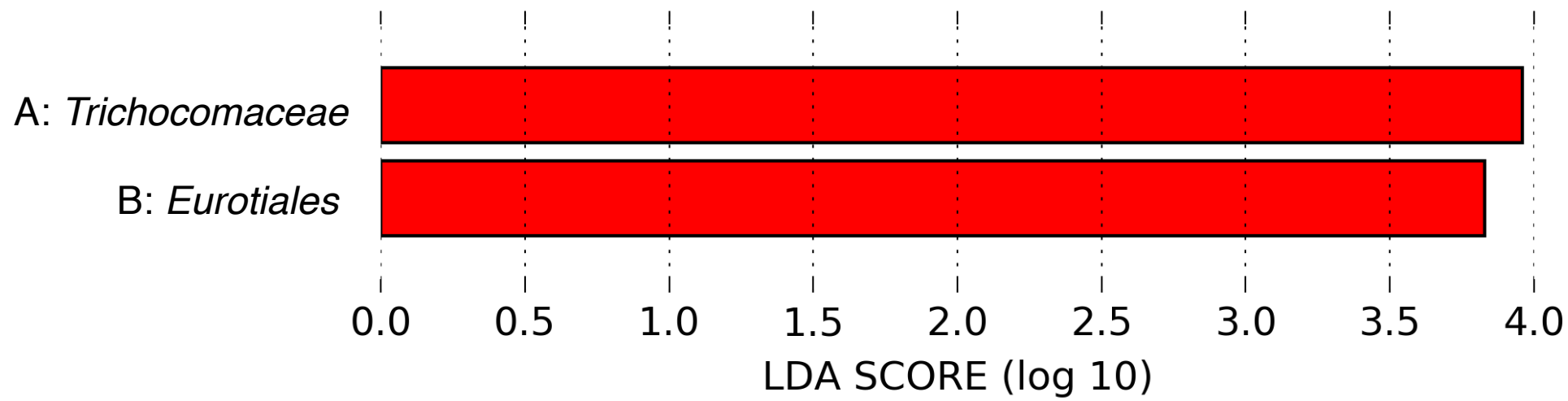

Supplement: Supplementary file 4 — Figure S3. LEfSe shown as a cladogram (a) and LDA score (b). The HIV-uninfected individuals are displayed in red. (PDF 224 kb) [file 12866_2018_1274_MOESM4_ESM.pdf]

**a** HIV-infected

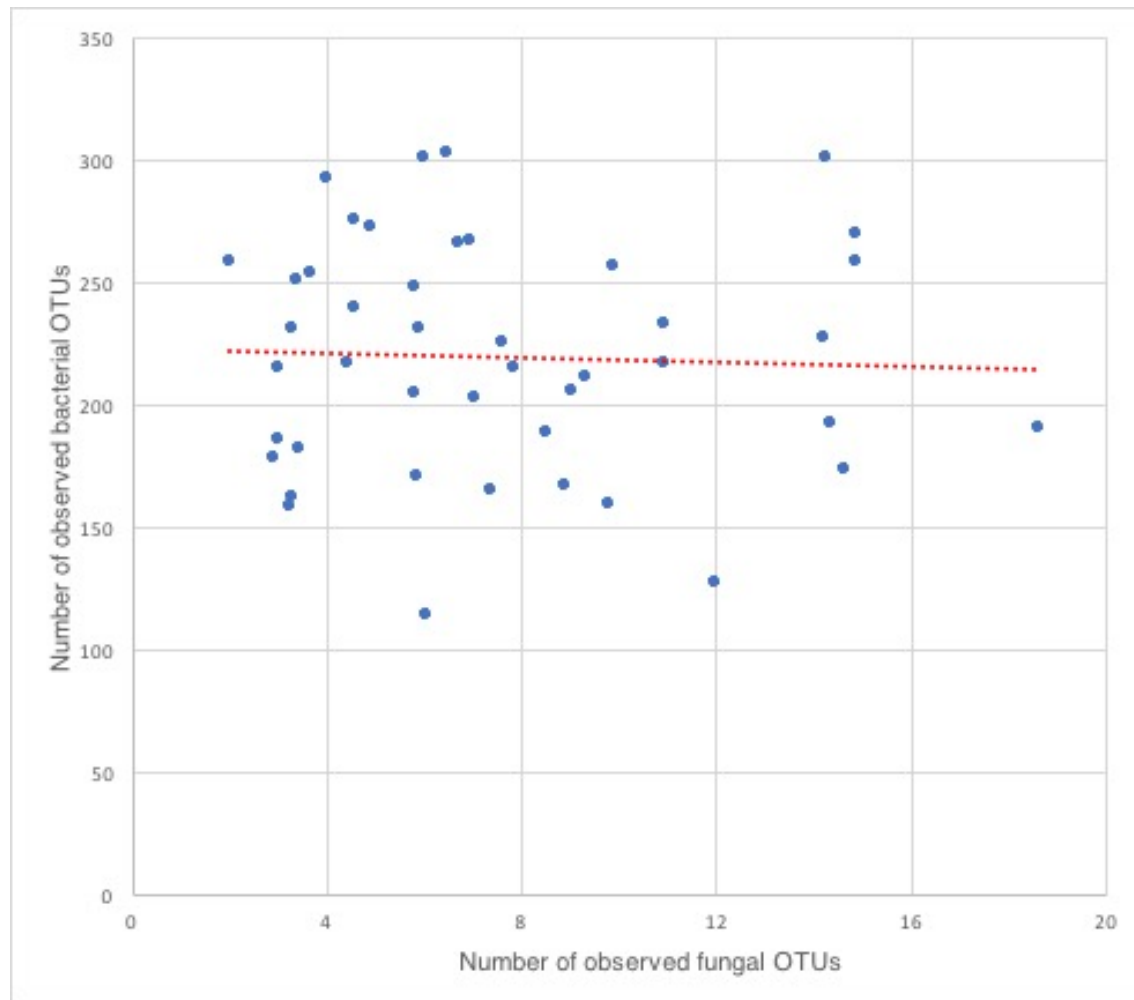

**b** HIV-uninfected

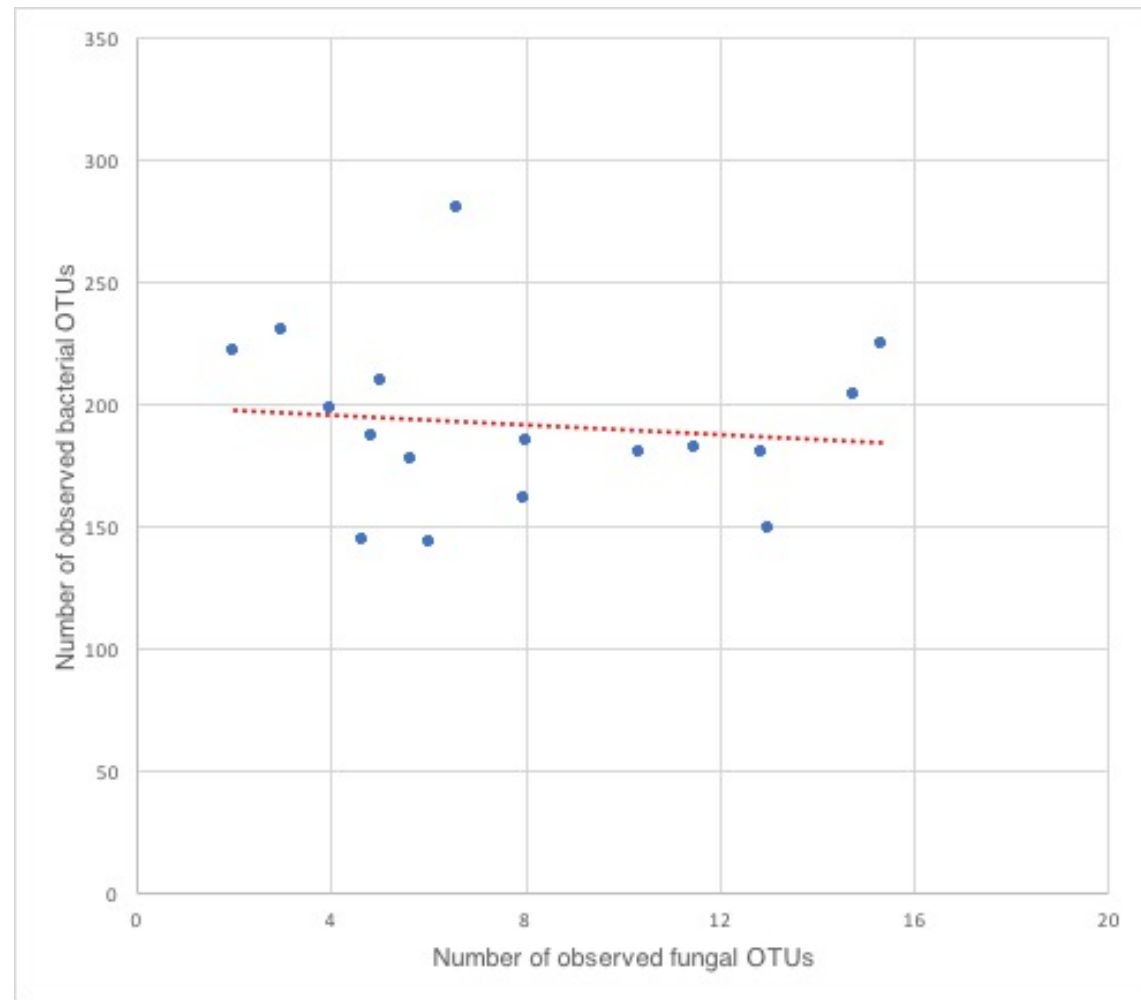

Supplement: Supplementary file 5 — Figure S4. Scatter plot showing the relationship between the number of observed bacterial and fungal OTUs in the HIV-infected (a) and HIV-uninfected individuals (b). (PDF 67 kb) [file 12866_2018_1274_MOESM5_ESM.pdf]

**a**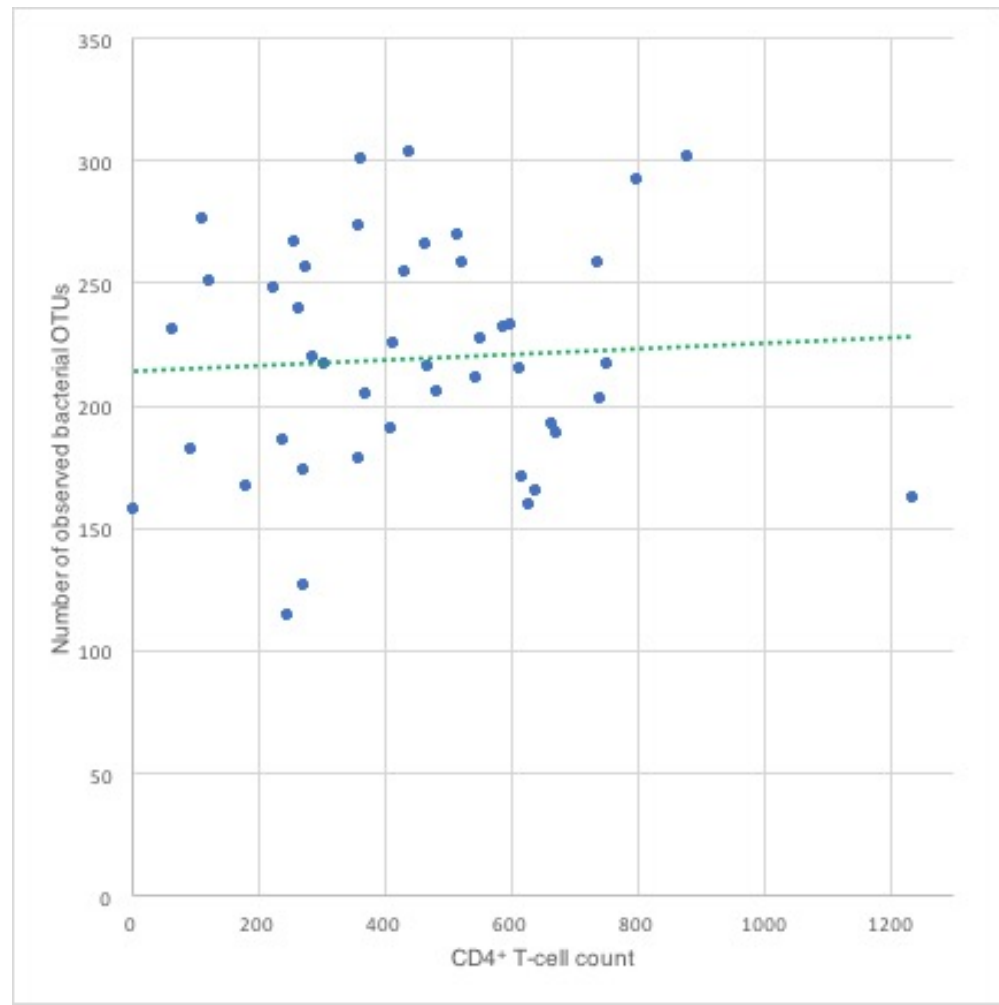**b**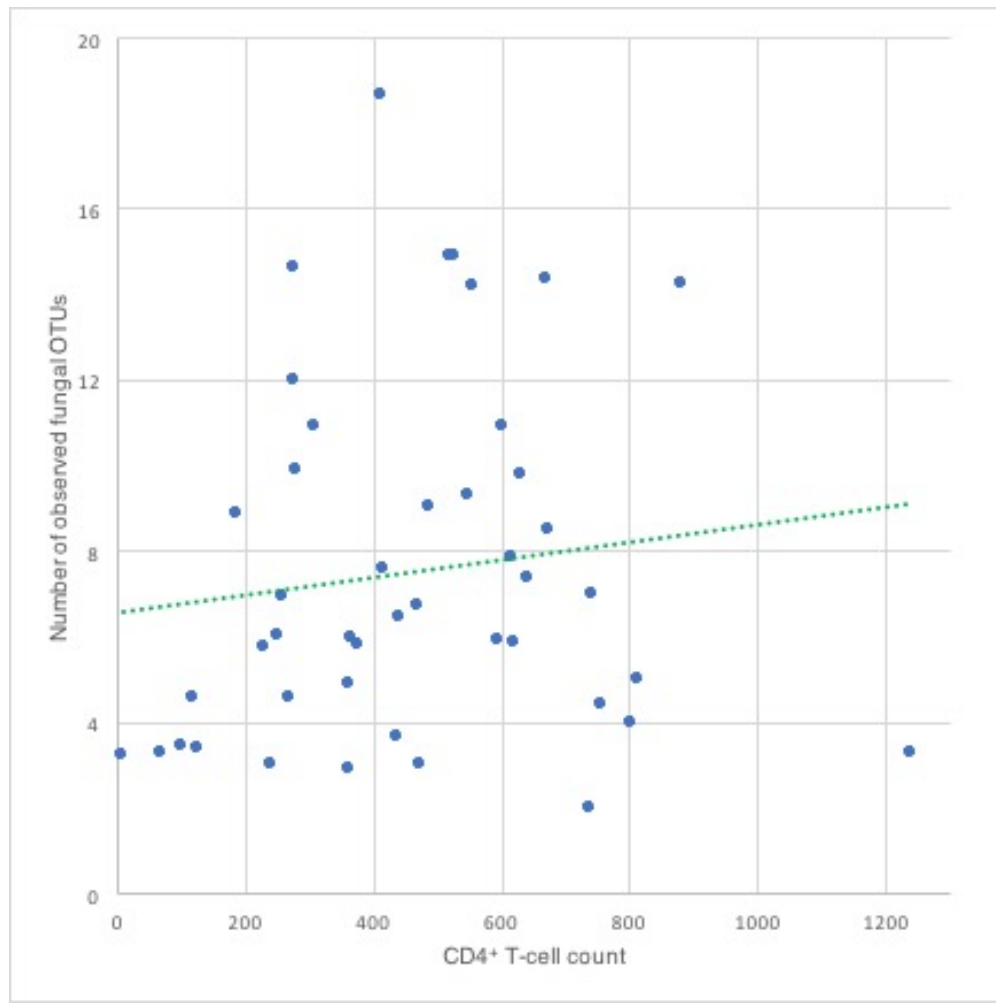

Supplement: Supplementary file 6 — Figure S5. Scatter plot showing the relationship between CD4+ T-cell counts and the number of observed bacterial (a) and fungal OTUs (b) in the HIV-infected individuals. (PDF 57 kb) [file 12866_2018_1274_MOESM6_ESM.pdf]
